# Clinical and genomic safety of treatment with *Ginkgo biloba L.* leaf extract (IDN 5933/Ginkgoselect®Plus) in elderly: a randomised placebo-controlled clinical trial [GiBiEx]

**DOI:** 10.1186/s12906-018-2080-5

**Published:** 2018-01-22

**Authors:** Stefano Bonassi, Giulia Prinzi, Palma Lamonaca, Patrizia Russo, Irene Paximadas, Giuseppe Rasoni, Raffaella Rossi, Marzia Ruggi, Salvatore Malandrino, Maria Sánchez-Flores, Vanessa Valdiglesias, Barbara Benassi, Francesca Pacchierotti, Paola Villani, Martina Panatta, Eugenia Cordelli

**Affiliations:** 1 Unit of Clinical and Molecular Epidemiology, IRCCS San Raffaele Pisana, San Raffaele University, Via di Val Cannuta 247, 00166 Rome, Italy; 2 Department of Human Sciences and Quality of Life Promotion, San Raffaele University, Via di Val Cannuta 247, 00166 Rome, Italy; 3 RSA San Raffaele Rocca di Papa, Via Ariccia, 16, 00040 Rocca di Papa (RM), Italy; 4 RSA San Raffaele Sabaudia, via Borgo Nuovo, 04010 Sabaudia (LT), Italy; 5 RSA San Raffaele Montecompatri, Via San Silvestro, 67, 00077 Montecompatri (RM), Italy; 6 Indena S.P.A., viale Ortles, 12, 20141 Milan, Italy; 7 DICOMOSA Group, Department of Psychology, Universidade de A Coruña, A Coruña, Spain; 8 Laboratory of Biosafety and Risk Assessment, ENEA CR Casaccia, Via Anguillarese, 301, 00123 Rome, Italy

**Keywords:** *Ginkgo biloba Extract*, Safety, Genomic stability, DNA cell maintenance

## Abstract

**Background:**

Numerous health benefits have been attributed to the *Ginkgo biloba* leaf extract (GBLE), one of the most extensively used phytopharmaceutical drugs worldwide. Recently, concerns of the safety of the extract have been raised after a report from US National Toxicology Program (NTP) claimed high doses of GBLE increased liver and thyroid cancer incidence in mice and rats. A safety study has been designed to assess, in a population of elderly residents in nursing homes, clinical and genomic risks associated to GBLE treatment.

**Methods:**

GiBiEx is a multicentre randomized clinical trial, placebo controlled, double blinded, which compared subjects randomized to twice-daily doses of either 120-mg of IDN 5933 (also known as Ginkgoselect®Plus) or to placebo for a 6-months period. IDN 5933 is extracted from dried leaves and contains 24.3% flavone glycosides and 6.1% of terpene lactones (2.9% bilobalide, 1.38% ginkgolide A, 0.66% ginkgolide B, 1.12% ginkgolide C) as determined by HPLC. The study was completed by 47 subjects, 20 in the placebo group and 27 in the treatment group. Clinical (adverse clinical effect and liver injury) and genomic (micronucleus frequency, comet assay, *c-myc*, *p53*, and *ctnnb1* expression profile in lymphocytes) endpoints were assessed at the start and at the end of the study.

**Results:**

No adverse clinical effects or increase of liver injury markers were reported in the treatment group. The frequency of micronuclei [Mean Ratio (MR) = 1.01, 95% Confidence Intervals (95% CI) 0.86–1.18), and DNA breaks (comet assay) (MR = 0.91; 95% CI 0.58–1.43), did not differ in the two study groups. No significant difference was found in the expression profile of the three genes investigated.

**Conclusions:**

None of the markers investigated revealed a higher risk in the treatment group, supporting the safety of IDN 5933 at doses prescribed and for duration of six months.

**Trial registration:**

ClinicalTrials.gov Identifier: NCT03004508, December 20, 2016. Trial retrospectively registered.

**Electronic supplementary material:**

The online version of this article (10.1186/s12906-018-2080-5) contains supplementary material, which is available to authorized users.

## Background

*Ginkgo biloba,* a slow-growing tree indigenous to Eastern Asia, is one of the oldest species of trees on the planet, with health-promoting properties and with a long history of use in herbal medicines [1–5]*.* In traditional medicine the use of leaves and seeds from the Ginkgo tree to treat respiratory and cardiovascular disorders, sexual dysfunction, and loss of hearing [6] dates back thousands of years. In Chinese medicine, *Ginkgo biloba* seeds have been used to treat pulmonary symptoms, alcohol abuse, and bladder infections, while ginkgo leaves came into use later to treat skin infections, as well as heart and lung disease [7, 8]*.*

Modern use of *Ginkgo biloba extract* is centered on leaf-based preparations and numerous health benefits have been attributed to its use. *Ginkgo biloba leaf extract* (GBLE) are commonly standardized to contain 24% of ginkgo flavone glycosides and 6% terpene lactones [2, 9]*.* Currently, GBLE is mostly utilized for the treatment of cognitive impairment [10]*.* GBLE is one of the most commercialized medicinal plants: in Europe it is principally regulated as herbal medicine, while in the US it is used as a dietary supplement, thus available in stores and online. The leaf extract is one of the five top-selling herbal supplements [11, 12]*.* The standardized extract is administered in Europe at a clinical daily dosage of 120–240 mg for at least 8 weeks [6, 13, 14]*.* Although the main use of the GBLE is to improve cognitive functions in elderly showing memory loss, the extract has been used to treat cardiovascular disease, idiopathic sudden sensorineural hearing loss, cerebral ischemia, and also to decrease chemotherapy-associated toxicity [3, 6, 15–17].

Different mechanisms of action for GBLE have been proposed. As far as memory loss is concerned the antioxidant effect, caspase-3–activation inhibition and the anti-amyloid aggregation are the most investigated. Other mechanisms involve antagonism of the platelet-activating factor by the ginkgo lids, antioxidant activity of the flavonoids attributed to scavenging of reactive oxygen species, chelation of metal ions, and increasing the concentration of superoxide dismutase and glutathione-*S*-transferase; antagonism of major inhibitory receptors of the central nervous system, glycine, and GABA receptors; modulation of neurotransmitter concentrations or receptor densities; reduction of NO release; inhibition of mitochondrial dysfunction; and modulation of P450 enzymes [4, 18–20]*.*

The extensive literature on the effect of treatment with GBLE in humans is quite contrasting. This is especially true in studies using Ginkgo to treat dementia or to prevent cognitive loss [21–24]*.* On the contrary, when the safety of treatment with GBLE is concerned, no consistent adverse effects were reported. In a review of studies on GBLE administered to humans, Diamond et al. concluded that adverse events, as described in case reports, occurred in patients that were taking additional medicines or had comorbid conditions [21]. A large randomized, double-blind clinical trial of GBLE efficacy in reducing dementia was conducted between 2000 and 2008 [25]. The authors found similar profiles of adverse events between the group taking twice-daily doses of 120 mg of GBLE and the placebo group. Rates of mortality and incidences of coronary heart disease, angina, angioplasty, or stroke were also similar between the two groups [21–24]. A review paper re-evaluated the results of human trials from 2008 to 2012 using GBLE to treat or to prevent Alzheimer’s disease [10]; this paper did not reveal differences in rates of adverse effects between the experimental and the placebo groups, similarly to another paper which showed no adverse effects at a daily dose of 240 mg of GBLE EGb 761 ® in the treatment of dementia [26]. However, a more recent meta-analysis suggests that more research is warranted to confirm the effectiveness and safety of GBLE in treating mild cognitive impairment and Alzheimer’s disease [27]*.*

In contrast with the substantial lack of adverse drug reaction which has characterized the clinical use of GBLE, a recent Technical Report, published by the US National Toxicology Program (NTP), evaluating toxicological and carcinogenic properties of the *Ginkgo biloba* extract (cas no. 90045–36-6) in F344/n rats and B6C3F1/n mice (gavage studies) concluded that there is evidence of carcinogenic activity of GBLE in rodents. The study reported an increased incidence of cancer of the thyroid gland in male and female rats and male mice, and of liver cancers in male and female mice. However the in vivo GBLE genotoxicity potential remained equivocal [28], and a more recent study reported negative results by comet assay in liver cells and by micronucleus assay in bone marrow of mice treated with GBLE [29]. After the publication of the NTP report, the Committee on Herbal Medicinal Products of the European Medicine Agency (EMA) [30] has issued documents assessing that, at present, there is no proof for an increased cancer risk in patients taking Ginkgo folium medicinal products at their approved posology, and the International Agency for Research on Cancer (IARC) on 2016 reported that there is inadequate evidence in humans for the carcinogenicity of *Ginkgo biloba* extract [31]. However, despite these documents from major international agencies, the results published by the NTP raised concerns, and planning clinical studies assessing the safety of GBLE treatment has become a priority.

## Methods

### Aim, design and setting of the study

The main aim of the present study is therefore to assess the clinical and genomic safety of the treatment with a common formulation of *Ginkgo biloba* L. leaf extract (IDN 5933 also known as Ginkgoselect®Plus, Indena S.p.A., Milan, Italy). The study design is a randomized clinical trial, placebo controlled, double blinded, performed in elderly institutionalized subjects. Given the obvious difficulty of assessing the risk associated to treatment using cancer incidence, we identified a battery of tests that may allow the detection of early events in the multistep process of carcinogenesis, especially as far as liver carcinogenesis is concerned. The first indexes to be evaluated were the presence of DNA damage and genomic instability, events both validated as precursors of cancer development. The comet assay and the micronucleus test in peripheral blood lymphocytes were evaluated for this purpose, respectively [32–36]*.* To provide a more specific assessment concerning events associated to hepato-carcinogenesis, the expression of some critical genes, i.e., c-*myb, p53*, and *ctnnb1* (β-catenin), reported to be modulated in early stages of carcinogenesis [37–39], was comparatively evaluated in treated subjects versus placebo. Finally, the presence of adverse clinical events, or the presence of liver injury associated to the experimental treatment was evaluated in all participants to the clinical trial.

The GiBiEx multicenter, randomized. Double blind, placebo-controlled clinical trial compared subjects receiving twice-daily doses of either 120-mg of *Ginkgo biloba* L. leaf extract (IDN 5933) or placebo for a 6 months period. A population of 140 elderly residents (≥ 65 years) was enrolled among the residents of three nursing homes of the San Raffaele network, i.e., the San Raffaele Montecompatri, the San Raffaele Rocca di Papa, and the San Raffaele Sabaudia, between June and November 2015. Patients with previous report of increased bleeding tendency receiving treatment with anticoagulant and antiplatelet drugs, with, cognitive impairment, refusing to sign the informed consent, and with a life expectancy of less than 1 year were not considered eligible for enrollment. Overall, 74 subjects didn’t meet the eligibility criteria and were excluded from the study. Eventually, 66 subjects (age > 65) signed the informed consent and accepted to participate to the study: 26 males 839.4%) and 40 females (60.6%). All participants were randomly allocated to 120 mg IDN 5933, twice per day in tablet form, or to placebo with a 1:1 ratio via computer-generated randomized allocation by an independent data management contractor with blocking by site.

The *Ginkgo biloba* leaf extract (IDN 5933 also reported as Ginkgoselect®Plus) utilized in this study was produced by INDENA S.p.A. Milan, Italy and contains 24.3% flavone glycosides and 6.1% of terpene lactones (2.9% bilobalide, 1.38% ginkgolide A, 0.66% ginkgolide B, 1.12% ginkgolide C) as determined by HPLC. Ginkgoselect®Plus is obtained by extracting the dried leaves of *Ginkgo biloba* using ethanol: water (70:30 *v*/v). The crude extract is then purified by subsequent concentration to water for the removal of biflavone compounds. Then two consecutive resin columns are used for the elimination of sugars and gingkolic acids respectively. The obtained product is then dried for residual ethanol and water elimination. Ginkgolic acids are considered unwanted substance of the extract and must be not more than 5 ppm (NMT 5 ppm). Only water and ethanol are used as solvents for the preparation of IDN 5933 (see Additional file 1).

At the start of the study (T0) all participants were administered a questionnaire concerning demographics characteristics, life-style, smoking habit, and relevant clinical history including concomitant treatments (see Additional file 2). A sample of 20 ml of blood was collected from each participant at T0 and at the end of the study (T1). The duration of the study was 6 months: 34 subjects received twice-daily doses of 120-mg of experimental treatment, *Ginkgo biloba* L. leaf extract IDN 5933; 32 subjects received placebo.

A total of 19 subjects (28.8%), withdrew from the study for the following reasons: death (1 subject), for acute pancreatitis in a chronic renal failure patient, discharge (10 subjects), admission to another hospital for worsening state of health (4 subjects), and discontinued treatment (4 subjects). The study was completed by 47 subjects (71.2%, 18 males and 29 females): 20 subjects in the placebo group (9 males and 11 females) and 27 subjects in the treatment group (9 males and 18 females).

Clinical conditions of all patients were monitored at the beginning, during the six months of treatment, and at the end of the study by the medical personnel of each nursing home, after receiving special training. Biological samples were collected at time T0 and at time T1 to evaluate liver injury [gamma-glutamyl transferase (γGT), alanine aminotransferase (ALT), aspartate aminotransferase, (AST)]. DNA damage and genomic instability [*Comet Assay* and *Micronucleus assay (MN*)]. A subgroup of 17 individuals (8 treated versus 9 placebos) was monitored to compare the expression patterns of genes putatively associated to early events of hepatic carcinogenesis, i.e., *p53, c-my*b and *ctnnb1*.

### Micronucleus assay

Blood samples from 47 subjects were obtained by venipuncture, collected in heparinised tubes, and then transported in cold (4 °C) to the laboratory where they were processed. All samples were coded and analyzed under blind conditions**.** The micronucleus assay (MN) was performed following the protocol previously described by Fenech [40, 41]. The cultures were established in duplicate by adding 0.5 ml of whole blood to 4.5 ml of culture medium: RPMI 1640 Medium Gibco™ (Thermo Fisher Scientific, MA USA) containing 15% heat inactivated fetal bovine serum Gibco™ (Thermo Fisher Scientific, MA USA), 1% phytohaemagglutinin Gibco™ (Thermo Fisher Scientific, MA USA), 1% L-glutamine (200 mM) (Biowest Europe, Nuaillé – France) and 1% penicillin (5000 U/ml)/streptomycin (5000 μg/ml) Gibco™ (Thermo Fisher Scientific, MA USA). Cells were incubated at 37 °C, 5% CO_2_. Cytochalasin-B (6 μg/ml) (Sigma-Aldrich S.r.l. Milan, Italy) was added at 44 h to prevent cytokinesis. After 72 h of incubation, cells were centrifugated, collected, and treated with a mild hypotonic solution (0.075 M KCl at 4 °C), and then centrifuged immediately and fixed in Carnoy’s solution [3:1 methanol–acetic glacial acid). Air dried slides were prepared and stained with 5 μg/ml 4, 6-diamidino-2-phenylindole (DAPI) (Sigma-Aldrich S.r.l. Milan, Italy).

Microscope analyses were performed by employing a fluorescence microscope Axio Imager Z2 microscope (Carl Zeiss Microscopy GmbH, Jena, Germany) equipped with a Automated SlideFeeder ×80, connected to motorized and controlling the microscope components for automated focusing, light source adjustment (for bright field imaging) and fluorescence filter changes. A minimum of 1000 binucleated cells were automatically scored to determine the number of MN and binucleated cells with MN (BN) with Metafer software (Carl Zeiss AxioImager.Z2, Jena, Germany). After the automated scan, the image gallery was visually reviewed by an experienced scorer, following criteria described by Fenech for MN and BN [32, 33, and 41].

### Alkaline comet assay

Lymphocytes were separated by gradient centrifugation of blood on LeucoSep. After washing pellet was resuspended in cryopreservation medium (Synth-a-Freeze® Thermo Fisher Scientific, Waltham, MA USA) and stored at −80 °C. Cryopreserved lymphocyte aliquots were thawed, immediately diluted in 5 ml PBS and centrifuged 5 min at 180 g to remove the cryopreservation medium. The pellet was resuspended in PBS and cell suspension was processed for the alkaline comet assay. The assay was performed essentially as previously described [42]. Two slides were prepared for each experimental point. After overnight lysis at 4 °C in lysing solution [2.5 M NaCl, 100 mM Na_2_EDTA, 10 mM Tris, [pH 10] containing 10% DMSO and 1% Triton X-100], slides were placed in electrophoresis buffer [300 mM NaOH, 1 mM Na_2_EDTA, pH >13], and left in the solution for 25 min at 4 °C. Electrophoresis was carried out at 4 °C for 25 min, 27 V (0.8 V/ cm) and 300 mA. Slides were then neutralized, fixed and air-dried. Before scoring, slides were stained with 12 μg/ml ethidium bromide (Sigma-Aldrich) and examined at 200× magnification with an Olympus fluorescence microscope. Slides were analyzed blindly with a computerized image analysis system (Delta Sistemi, Italy). For each sample, 150 cells were analyzed from 2 different slides; the percentage of DNA in the tail of the comet (% TI) was used as the parameter for evaluation of DNA damage. Heavily damaged cells, i.e. hedgehogs (as determined by visual scoring or cells having more than 80% DNA in the tail), were not included in the measurement but they were counted and their percentage was calculated per sample.

### Gene expression of c-myb, p53, and ctnnb1

An aliquot of lymphocytes isolated as described above, was centrifuged and pellet was cryopreserved at −80 °C. Total RNA was extracted from blood samples by Trizol® (Invitrogen, Thermo Fisher Scientific, and Waltham, MA USA) protocol. The amount and purity of the extracted RNA was evaluated by fiber optic spectrophotometer (Nanodrop ND-1000, NanoDrop Technologies, Wilmington, DE, USA) calculating the 230/260 and 260/280 absorbance ratios. Two hundred nanograms of total RNA were retro-transcribed into total cDNA by TaqMan® Reverse Transcription Reagent (Applied Biosystems, Thermo Fisher Scientific), according to manufactures’ indications. Potential contamination by genomic DNA was verified by amplifying the human *β-actin* gene (Fw primer: 5′ TGA CGG GGT CAC CCA CAC TGT GCC CAT CTA 3′; rev primer: 5’ CTA GAA GCA TTT GCG GTG GAC GAT GGA GGG 3′) in a conventional thermocycler according to the following Polymerase Chain Reaction (PCR) conditions: one cycle at 95 °C (10 min), 40 cycles at 95 °C(30 s)-58 °C (30 s)-72 °C (30 s), one cycle at 72 °C (5 min). Reactions were run in 20 μl total volume by Taq Polymerase (Euroclone, Milan, Italy); PCR products (663 bp amplicon for the cDNA, 867 bp amplicon for the genomic DNA) were visualized on 1.5% agarose gel by ethidium bromide staining.

Analysis of the human (hsa) *c-myb, p53* and *ctnntb1* genes expression was carried out with 1 μL of cDNA using SYBR Green master mix (Applied Biosystems) and analyzed on a Eco™ Real-Time PCR System (Illumina, San Diego, CA, USA). All reactions were run in quadruplicate and the relative abundance of the specific mRNA level was calculated by normalizing to the GAPDH expression using the 2-ΔΔCt method [43]. Primers sequence is as follows:

- hsa_c-myb Fw 5’ ACCATGACTATGATGGGCTGC 3′
- hsa_c-myb Rev. 5’ TCCCCAAGTGACGCTTTCC 3′
- hsa_p53 Fw 5’ TAACAGTTCCTGCATGGGCGGC 3′
- hsa_p53 Rev. 5’ AGGACAGGCACAAACACGCACC 3′
- hsa_ctnnb1 Fw 5’ AGCTTCCAGACACGCTATCAT 3′
- hsa_ctnnb1 Rev. 5’ CGGTACAACGAGCTGTTTCTAC 3′
- hsa_GAPDH Fw 5’GAG TCA ACG GAT TTG GTC GT3’
- hsa_GAPDH Rev. 5’ GAC AAG CTT CCC GTT CTC AG 3′

Data were analyzed in each sample in terms of increase/decrease of the specific gene expression at T1 compared to T0, with a cut-off value ≥ 2-fold change.

### Statistical analysis

A pilot study with this sample size will identify with 95% confidence a problem with a 5% probability to occur in a potential study participant [44]. Descriptive statistics were measured for all patients’ characteristics. The presence of heterogeneity between groups was tested with the Chi-square test for qualitative variables and with the Students T-test for continuous variables. Logarithmic transformation of data was applied when necessary to normalize the distribution. Several variables were categorized before analysis, e.g., marital status, education, weight, height, smoking habit, comorbidities, and liver function tests. The comparison of clinical and molecular indexes of DNA damage in the two groups of treatment, were evaluated with univariate analysis, and subsequently with multiple regression analysis, using general linear models, to take into account the influence of age, sex, smoking status, and other covariates as mental and behavioral disorders.

Statistical analysis concerning the MN assay evaluated the frequency of micronucleated cells per 1000 binucleated cells. To take into account the large overdispersion, due to the random nature of variables, a mixed distribution was applied to fit regression models, i.e. the Poisson and Negative Binomial Models. A lognormal model was applied to the fit Tail intensity in the comet assay.

In the Poisson and Negative Binomial Models, a specific analysis was used (through differences in starting values and convergence criteria of the algorithms) to deal with the overdispersion, scaling the standard errors using the square root of the Pearson chi-square dispersion. The standard errors were adjusted to compensate for the overdispersion in both methods. The parameters estimation was accomplished via maximum likelihood, and the AIC - Akaike’s Information Criterion [45] was taken into account to select the best model. For each model the Mean Ratio (MR), together with asymptotic 95% confidence intervals (95% CI) was computed. Each model included age, sex and smoking habit, and actual confounders.

The critical limit for significance was set at *p* < 0.05. The statistical softwares used for the analysis were Stata [46], and SPSS (Statistical Package for Social Science, Version 16.0) [47].

## Results

The descriptive statistics of the 47 individuals who completed the clinical trial are reported in Table 1. The number of subjects in the treatment arm was slightly higher (27 vs 20), but none of the variables taken into consideration, including age, sex, marital status, height, weight, education, and smoking habit significantly differed between the two groups. A specific analysis concerning the 19 withdrawers revealed that in this group statistics concerning the variables reported in Table 1 did not differ from those of subjects completing the protocol. The large majority of study subjects were affected by chronic conditions. The most frequent were diseases of the circulatory system (59.6%), diseases of the nervous system (55.3%), mental and behavioral disorders (55.3%), endocrine, nutritional and metabolic diseases (29.8%), and diseases of the respiratory system (25.5%). Multimorbidity was a common feature. The prevalence of chronic diseases did not significantly differ between subjects randomized to the treatment with IDN 5933 or to placebo.

**Table 1:** Distribution of participants to the GIBIEX study according to main individual characteristics at enrollment

Characteristics	N	IDN 5933	Placebo	*p*-value^*^
	47	27 [57.5%]	20 [42.5%]	–
Age [Mean ± SD]	47	79.2 ± 10.7	81.2 ± 10.3	NS
Age Tertiles [%]	47			NS
< 77 years	17	11 [40.7%]	6 [30.0%]	
78–85	15	8 [29.6%]	7 [35.0%]	
≥ 86	15	8 [29.6%]	7 [35.0%]	
Sex [% Male]	18	9 [33.3%]	9 [45.0%]	NS
Marital Status [%]	43			NS
Unmarried	11	8 [32.0%]	3 [16.7%]	
Married	9	5 [20.0%]	4 [22.2%]	
Divorced	1	1 [4.0%]	–	
Widower	22	11 [44.0%]	11 [61.1%]	
Education [%]	35			NS
No degree/Elementary	27	13 [76.5%]	14 [77.8%]	
Middle School	4	–	4 [22.2%]	
High School/University/Other	4	4 [23.5%]	–	
Current Weight Kg [Mean ± SD]	38	68.3 ± 12.8	67.9 ± 18.9	NS
Usual Weight Kg [Mean ± SD]	32	70.4 ± 10.6	67.5 ± 13.5	NS
Height mt. [Mean ± SD]	40	1.64 ± 0.1	1.63 ± 0.1	NS
Smoking habit [%]	47			NS
Never Smoker	20	13 [48.2%]	7 [35.0%]	
Current Smoker	7	4 [14.8%]	3 [15.0%]	
Ex-smoker	12	7 [25.9%]	5 [25.0%]	
Not responding	8	3 [11.1%]	5 [25.0%]	

^*^t-test quantitative [for unequal variances] or Chi square qualitative [NS *p* > 0.05]

The presence of adverse clinical effects was evaluated recording all symptoms that could have been attributed to the study treatments. No specific symptoms were reported for any subjects in both arms. In the first week of the study a subject deceased, for acute pancreatitis, in a subject affected by chronic renal failure: after breaking randomization code and realizing the subject was under treatment with IDN 5933 extract we communicated the death to ethics committees which evaluated the protocol and to the National Health Institute (Istituto Superiore di Sanità: ISS). The death was attributed by the medical personnel of the nursing home to the worsening of an already compromised multipathological condition, and that no association could be hypothesized with the treatment with IDN 5933. The other clinical index evaluated in the study group considered the level of liver enzymes (γGT, ALT, AST, and alkaline phosphatase) which have been shown to be positively associated with hepatocellular carcinoma risk [48].

Table 2 shows the distribution of subjects with pathological values of most common tests of liver injury, i.e., AST, ALT, and γGT. The occurrence of subjects with elevated values of AST and ALT was very low overall, with only one subject showing elevated values for both markers (3.7%). Elevation of γGT was more common with 6 subjects overall, 4 in the treated group and 2 in the placebo. The same numbers were found at the beginning of the study (T0) and at the end (T1).

**Table 2:** Descriptive analysis of liver function tests in GIBIEX Study. Stratification of pathological vs normal reports by group of treatment

Liver function test	IDN 5933 N 27	Placebo N 20
Aspartate Aminotransferase [AST]		
T0		
Normal	26	20
Pathological	1	0
T1		
Normal	27	20
Pathological	0	0
Alanine Aminotransferase [ALT]		
T0		
Normal	26	20
Pathological	1	
T1		
Normal	27	20
Pathological	0	0
Gamma Glutamyl Transferase [γGT]		
T0		
Normal	23	18
Pathological	4	2
T1		
Normal	23	18
Pathological	4	2

The level of genomic instability in the study group has been evaluated performing the cytokinesis block micronucleus test in peripheral blood lymphocytes. The main study results, summarized in Table 3, show no difference when the frequency of MN in the IDN 5933 group is compared with the placebo, i.e., 12.08‰ ± 6.1 vs 12.68‰ ± 7.1 (see Additional file 3). To allow an intrinsic validation of MN assay results, MN frequency was evaluated also in relation to other critical variables. As constantly reported by the literature, a lower frequency – though not significantly so - was found in males (in the treated group) and in current smokers in both placebo and treated subjects. To take into account the role of confounding variables, a Poisson regression analysis was performed, which included age, sex, and smoking habit plus actual confounders (mental and behavioral disorders). Subjects treated with IDN 5933 reported an adjusted frequency of MN that was substantially equivalent to that of placebo (MR = 1.01, 95% CI 0.86–1.18).

**Table 3:** Micronucleus frequency [Mean ± SD]according to main individual characteristics of participants to the GIBIEX study [values at T0]

Characteristics	N	IDN 5933 [MN‰]	N	Placebo [MN‰]	*p*-value^*^
MN‰ T0	27	10.00 ± 5.32	20	9.84 ± 4.95	
					NS
MN‰ T1	27	12.08 ± 6.11	20	12.68 ± 7.07	
AgeTertiles					
65–77 years	11	11.91 ± 6.75	6	8.10 ± 3.35	NS
78–85	8	8.27 ± 3.11	7	10.84 ± 3.85	
≥ 86	8	9.10 ± 4.53	7	10.33 ± 6.99	
Sex					
Male	9	10.22 ± 4.55	9	8.73 ± 5.13	NS
Female	18	9.89 ± 5.79	11	10.74 ± 4.84	
Marital Status					
Unmarried	8	8.97 ± 4.37	3	5.50 ± 2.02	NS
Married	5	9.29 ± 3.25	4	8.81 ± 3.31	
Divorced	1	6.11 ± −	–		
Widower	11	11.44 ± 7.02	11	11.67 ± 5.57	
Education					
No degree/Elementary	13	11.61 ± 6.78	14	9.93 ± 5.37	NS
Middle School	–	–	4	10.06 ± 4.75	
High School/University	4	10.31 ± 3.85	–	–	
Smoking habit					
Never	13	9.28 ± 5.67	7	9.01 ± 3.56	NS
Current Smoker	4	8.93 ± 5.52	3	8.25 ± 2.99	
Ex-smoker	7	10.82 ± 4.86	5	11.44 ± 6.06	
Not responding	3	12.59 ± 6.39	5	10.34 ± 7.05	

^*^t-test quantitative [for unequal variances] or Chi square qualitative [NS: p > 0.05]. Row data are shown in Additional file 3

The parallel analysis aimed at evaluating the association of IDN 5933 treatment with DNA damage as assessed by comet assay was performed using the % of DNA in the tail (TI%) as unique endpoint, after performing sensitivity analyses which did not find meaningful differences among the other parameters describing DNA damage (tail moment and tail length) (Table 4). Subjects treated show a mean value at the end of the study of 6.03% ± 4, 06, not significantly different from the correspondent value of 7.15% ± 4.43 in the placebo group (see Additional file 4). As for the MN assay, comet assay data were examined in relation to other variables considered in the study. No difference was observed by sex, by age, or by level of education. Current smokers showed a much lower intensity of DNA damage, although the small numbers did not allow having a proper statistical evaluation of this difference. Also for the comet assay, a multiple regression analysis was performed to take into account confounding. A log normal model, adjusted for age, sex, and smoking habit showed a mean ratio of 0.91 for IDN 5933 treated subjects when compared to placebo, (95% CI 0.58–1.43). The only significant result was the positive trend by age, with a genetic damage which - in slight contrast with univariate data - increases by 3% each year of age (95% CI 1–5).

**Table 4:** DNA damage [Mean ± SD]according to main individual characteristics of participants to the GIBIEX study [values at T0]

Characteristics	N	IDN 5933 [TI%]	N	Placebo [TI%]	*p*-value^*^
TI% T0	27	7.12 ± 4.72	20	8.30 ± 3.83	
					NS
TI% T1	27	6.03 ± 4.06	20	7.15 ± 4.43	
AgeTertiles					
65–77 y.	11	8.15 ± 6.51	6	7.33 ± 2.87	NS
78–85 y.	8	6.23 ± 3.09	7	9.51 ± 4.83	
≥ 86 y.	8	6.59 ± 3.16	7	7.94 ± 3.67	
Sex					
Male	9	9.31 ± 4.78	9	7.06 ± 2.28	NS
Female	18	6.03 ± 4.43	11	9.32 ± 4.60	
Marital Status					
Unmarried	8	7.92 ± 6.35	3	8.42 ± 5.23	NS
Married	5	6.72 ± 3.82	4	5.66 ± 2.36	
Divorced	1	6.07 ± 0	–	–	
Widower	11	7.39 ± 4.61	11	9.63 ± 3.88	
Education					
No qualify/Elementary	13	8.52 ± 5.22	14	8.45 ± 4.01	NS
Middle School	–	–	4	8.70 ± 4.06	
High School/University	4	3.97 ± 1.60	–	–	
Smoking habit					
Never	13	6.91 ± 4.27	7	11.32 ± 3.79	NS
Current Smoker	4	4.77 ± 1.36	3	6.35 ± 2.51	
Ex-smoker	7	9.52 ± 6.52	5	8.36 ± 3.63	
Not responding	3	5.57 ± 3.91	5	5.19 ± 0.99	

^*^t-test quantitative [for unequal variances] or Chi square qualitative [NS: p > 0.05]. Row data are shown in Additional file 4

The last index of safety that was investigated is the regulation of the expression of genes that have been reported to be involved in liver carcinogenesis. Given the supervised approach to data analysis, and the emphasis on the up or down regulation of gene expression, we evaluated the distribution of subjects with significantly altered profiles for the candidate genes with the Fisher’s exact test of independence. The expression level of *ctnnb1* could not be detected in most samples; gene expression could be measured in only 5 subjects (2 IDN 5933 and 3 placebos) out of 17. All of them reported a down-regulation of *ctnnb1* in T1 when compared to T0 (see Additional file 5). As regards *c-myb* and *p53*, no significant difference (*p* < 0.05 parametric and non parametric assays) was observed in the distribution of subjects with up- and down-regulated levels of gene expression (see Additional file 5).

A summary of the different indexes used to assess the safety of IDN 5933 treatment is reported in Table 5. All indexes evaluated in the clinical trial were listed and the results summarized and briefly commented.

**Table 5:** Synthesis of main results concerning the assessment of clinical and genomic safety of subjects treated with IDN 5933

Safety threat	Assessment	Marker	Results	Interpretation
Clinical adverse effects	Diary of adverse effects / clinical records at T0 and T1	Treatment associated symptoms / Unexpected symptoms	1 death unrelated to treatment No symptoms associated to treatment	No occurrence of clinical symptoms associated to the treatment
Liver injury	Laboratory testing at T0 and T1	ALT – AST – γGT	No new subjects reported pathological exams at T1	No liver injury associated to the treatment
Genomic instability	Laboratory testing at T0 and T1	Cytokinesis Block Micronucleus assay	No increase of mean MN frequency in subjects treated with IDN 5933 [MR = 0.98; 0.84–1.16]	No increase of genomic instability associated to the treatment
DNA damage	Laboratory testing at T0 and T1	Comet assay	No increase of mean TI% damage in subjects treated with IDN 5933 [MR = 0.96; 0.64–1.40]	No increase of DNA damage associate to the treatment
Modulation of genes involved in liver carcinogenesis	Laboratory testing at T0 and T1	*c-myb – p53 – ctnnb1* expression level [mRNA level]	No association with treatment	No altered expression of liver cancer genes associated to the treatment

## Discussion

The existing discrepancy between international agencies in the classification of the GBLE makes the use of definitions and the choice of the proper regulation quite difficult. While safety studies of drugs already on the market are thoroughly regulated, and literature about post-authorization safety study (PASS) is extensive [49], the use of herbs and supplements as complementary health approaches is not regulated by national and international agencies, and reports about safety of these products is mostly anecdotic or limited to observational studies. This lack of information has relevance for public health whenever a dietary supplement reaches the market, especially if, like in the case of the GBLE, millions of individuals assume regularly these extracts. The present study was designed applying to the IDN 5933 the same approach used for authorized medicinal product in PASS, with the aim of identifying, characterizing or quantifying safety hazards [50]. The present study was especially designed to check the effects of a mid-term treatment with IDN 5933 at therapeutic doses on genomic instability, DNA damage, and on the modulation of genes typically associated to early stages of carcinogenesis. The Randomized Clinical Trial (RCT) design allowed also to evaluate the occurrence of adverse clinical symptoms (especially gastrointestinal) - though the study is clearly underpowered - and other clinical features, such as the occurrence of altered hepatic exams.

The main concerns generated by the NTP study [28] referred to the excess of hepatocellular carcinoma and thyroid cancer in animals exposed to high doses of GBLE. The difficulty to identify a population with long history of treatment with GBLE and the low frequency and the long latency of these tumors, especially the first, make nearly impossible to design an epidemiological study using cancer incidence as the primary endpoint. Therefore, we planned a study evaluating different indexes that may predict the risk of developing cancer in subjects treated at therapeutic doses with the IDN 5933. The frequency of micronucleated cells in binucleated lymphocytes is a well-known marker of DNA damage and genomic instability [32]. Furthermore, the high frequency of this marker in healthy subjects has been associated to cancer risk, nearly doubling cancer incidence in subjects in the highest tertile of MN distribution [33]. In addition to the MN test we also applied comet assay to assess DNA strand breaks in circulating lymphocytes. Although an association between increased level of DNA damage as assessed by comet assay, and cancer risk has not been proven jet, the test is widely applied in human biomonitoring studies to measure DNA damage as a marker of exposure to genotoxic agents [35]. Moreover, its ability to discriminate chemical carcinogens and non-carcinogens in rodents has been demonstrated [51, 52] and the Organization for Economic Co-operation and Development (OECD) recently included the alkaline comet assay in rodents among validated tests useful to identify substances that cause DNA damage [53]. As regards the possible criticism that the duration of the trial should have been longer, we have consider that the outcome of this safety study was not the possible carcinogenicity of GBE, but the early events of genomics risks. As regards the micronucleus and the comet assays, there is extensive literature that even an exposure of few days may alter the background frequency of DNA damage.

Finally, an over-expression of genes involved in the liver cancer pathway has been reported after the NTP study by Hoenerhoff and colleagues [39]. To provide a picture of the expression pattern of genes acting in the early stages of liver carcinogenesis, we monitored the expression of *ctnntb1*, a gene often impaired in human hepato-carcinogenesis, which seemed to be specifically involved in liver tumors occurring in GBLE-treated mice [39], as well as a cancer-specific *p53* onco-suppressor gene and the *c-myb* oncogene. Under our experimental conditions, the comparison of the gene expression pattern at the beginning and at the end of the study period did not reveal any IDN 5933-specific modulation.

As far as adverse clinical effects are concerned, a large battery of symptoms has-been described and potentially attributed to treatment with GBLE [54]. The present RCT was not specifically designed to test clinical safety of treatment with IDN 5933 neither had the statistical power to detect possible adverse effect occurring at a frequency similar to that reported in the literature. However, to provide new informations we decided to perform the study in a group of elderly subjects (≥ 65 years), who not only are the most common users of supplements, but also are more sensitive to genomic damage and to functional failure. The absence of adverse clinical symptoms, together with the unaffected hepatic functionality provides a piece of information supporting clinical safety of IDN 5933 mid-term treatment in elderly, although the small size of the study does not allow excluding rarer adverse effects.

The present study was specifically designed to test the presence of DNA damage and genomic instability in elderly subjects treated with IDN 5933. The lack of association with IDN 5933 treatment reported for both these biomarkers is well circumstantiated, and also the possible role of clinical and epidemiological confounders has been considered with multiple regression modeling, Furthermore, the RCT design offers a critical advantage, removing through randomization most of the limitations which affect human studies using biomarkers like the MN assay or the comet assay.

Measuring gene expression profiles of genes which have been putatively involved in the origin of liver tumors in GBLE-treated mice is an original approach to measure early events of the carcinogenesis pathway eventually leading to liver carcinogenesis. The choice of liver specific and unspecific genes provides a meaningful description of gene activation, and - besides the obvious limitations due to the use of surrogate cells - of the potential impact of GBLE treatment on cancer risk.

### Strengths and limitations of the study

The present study has a number of strengths. The most important is the multiendpoint approach, which provides an overview of most critical hazards putatively associated to GBLE treatment. Another major pro is the study design. RCT’s are the most reliable and bias proof studies, with randomization and double blindness providing credible results. Furthermore, the condition of resident in a nursing home determined a standardization of the diet, removing one of the confounding factors most difficult to control for. Finally, the choice of a population of elderly subjects affected by various chronic conditions, offers new data on a group which is generally not considered in clinical trials, mostly based on younger and healthier people [55]. As regards the weaknesses of the study, the most evident is the small size of the study and the large number of withdrawals. Elderly living in nursing homes are quite often affected by cognitive impairment, and this condition, together with - at a much lower extent - a life expectancy of less than one year determined a large reduction of subjects eligible for enrolment. Some families invited their relative to deny the consent to participate to the study because of information mostly coming from the internet about unspecified risks reported for the GBLE. The large number of subjects (19 subjects) withdrawing the study is explained by the advanced age and by the residency in a private nursing home. An external event that caused several requests of discharge was a cut of the government support for indigent families. The absence of contacts with these subjects did not allow performing an intention-to-treat analysis of data, although all sensitive analyses performed in this subgroup on study variables at T0 did not show any difference with those patients who continued the study.

## Conclusion

The results of this study show that the multiple endpoint design is an efficient approach to test safety of drugs/supplements, whenever a combination of clinical and genomic hazards is suspected. In addition, the choice of a study population over 65 years of age increases the possibility to reveal adverse effects associated to treatments, although the higher probability of withdrawal recommend to recruit larger study population.

In conclusion, as summarized by Table 5, none of the markers measured in the present clinical trial revealed a higher risk in the experimental group, supporting the safety of treatment with IDN 5933 at doses prescribed and for duration of six months.

## Additional files

Additional file 1: Certificate. Analysis Certificate of IDN 5933/Ginkgoselect®Plus and placebo [LM42506]. Indena S.p.A. declares all components used for the preparation of products. (PDF 622 kb)

Additional file 2: Questionnaire. GiBiEx QUESTIONNAIRE. The English version of the questionnaire administered to all participants to the GiBiEx study is reported. (PDF 10 kb)

Additional file 3: Comet Assay Raw data. Individual data relative to comet assay. For each donor data are shown as before (T0) and after (T1) placebo or IDN 5933 administration. (PDF 425 kb)

Additional file 4: Gene expression Raw data. Data were analyzed in each sample in terms of increase/decrease of the specific gene expression at T1 compared to T0, with a cut-off value ≥2-fold change (The values reported represent the excel conversion of the original raw data (average Ct calculated out of four replicates loaded onto the 48-wells PCR plate); the raw data are produced as “.csv” file by the EcoIllumina software and need to be converted into excel file for delta delta Ct analysis). (PDF 163 kb)

Additional file 5: Micronucleus Assay Raw data. Individual data relative to the MN Assay. Data are reported as before (T0) and after (T1) placebo or IDN 5933 administration. (PDF 683 kb)

## Abbreviations

ALT: Alanine aminotransferase
AST: Aspartate aminotransferase
BN: Binucleated cells with MN
CI: Confidence intervals
EMA: European Medicine Agency
GBLE: *Ginkgo biloba* leaf extract
GiBiEx: Randomised placebo-controlled clinical trial
IARC: International Agency For Research On Cancer
ISS: Istituto Superiore Di Sanità
MN: Micronucleus assay
MR: Mean ratio
NMT: Not more than
NTP: US National Toxicology Program
OECD: Organization For Economic Co-Operation And Development
PASS: Post-authorization safety study
RCT: Randomized clinical trial
SPSS: Statistical Package For Social Science
γGT: Gamma-glutamyl transferase
