# Supplementary material for: Clinical and genomic safety of treatment with Ginkgo biloba L. leaf extract (IDN 5933/Ginkgoselect®Plus) in elderly: a randomised placebo-controlled clinical trial [GiBiEx]
Source: BMC Complement Altern Med. 2018 Jan 22;18:22. doi: 10.1186/s12906-018-2080-5 (PMC5778811; doi:10.1186/s12906-018-2080-5)
Supplement: Supplementary file 1 — Certificate. Analysis Certificate of IDN 5933/Ginkgoselect®Plus and placebo [LM42506]. Indena S.p.A. declares all components used for the preparation of products. (PDF 622 kb) [file 12906_2018_2080_MOESM1_ESM.pdf]

indena

SEDE LEGALE: V.le Ortles, 12 – Milano (ITALIA)
STABILIMENTO: V.le Ortles, 12 – Milano (ITALIA)
TEL. +39 02 57496.1

Analysis Certificate N. 15/17/CQMI
Code : LM42506
Batch N. 89077
Preparation date: March 2015

PRODUCT: Ginkgo Biloba ethanolic extract film-coated tablets (placebo)

DETERMINATIONS	RESULTS	M. U.
**APPEARANCE**<br>9 mm diameter, white convex round shape film coated tablets	Complies	
**MEAN WEIGHT**	455.5	mg/tab.
**UNIFORMITY OF MASS**<br>Eur. Pharm. (2.9.5)	Complies	
**DISINTEGRATION TEST**<br>Eur. Pharm. (2.9.1)	< 30	min.
**HPLC IDENTIFICATION**<br>of Terpenes	Negative	
**HPLC IDENTIFICATION**<br>of Ginkgoflavonglucosides	Negative	
**As**<br>**Pb**<br>**Cd**<br>**Hg**	0.012<br>0.039<br>0.005<br>< 0.0005	ppm<br>ppm<br>ppm<br>ppm
**MICROBIOLOGICAL CONTROL**<br>Eur. Pharm. (2.6.12, 2.6.13 and 2.6.31)<br><br>- **Total Aerobic Microbial Count**<br>- **Total Combined Yeast/Moulds Count**<br>- **Bile-Tolerant Gram-Negative Bacteria**<br>- **Escherichia coli**<br>- **Salmonella**<br>- **Staphylococcus aureus**	 <br><br>500<br>< 10<br>absent<br>absent<br>absent<br>absent	 <br><br>cfu/g<br>cfu/g<br>cfu/g<br>/g<br>/25g<br>/g

Milan, March 31, 2015

Dr.ssa Viviana Cerutti
Head of Quality Control
INDENA S.p.A., Milan Plant

Franco Santini

INDENA S.p.A.
MILAN PLANT
TECHNICAL DIRECTOR
Dr. Franco Santini

indena

SEDE LEGALE: V.le Ortles, 12 – Milano (ITALIA)
STABILIMENTO: V.le Ortles, 12 – Milano (ITALIA)
TEL. +39 02 57496.1

Analysis Certificate N. 15/18/CQMI
Code : LM42502
Batch N. 89078
Preparation date: March 2015

PRODUCT: Ginkgo Biloba ethanolic extract 120 mg film coated tablets

DETERMINATIONS	RESULTS	M. U.
**APPEARANCE**<br>9 mm diameter, white convex round shape film coated tablets	Complies	
**MEAN WEIGHT**	414.6	mg/tab.
**UNIFORMITY OF MASS**<br>Eur. Pharm. (2.9.5)	Complies	
**DISINTEGRATION TEST**<br>Eur. Pharm. (2.9.1)	< 30	min.
**HPLC IDENTIFICATION**<br>of Terpenes	Positive	
**HPLC IDENTIFICATION**<br>of Ginkgoflavonglucosides	Positive	
**HPLC ASSAY**<br>of Bilobalide	3.44	mg/tab.
**HPLC ASSAY**<br>of Gingkolides A, B, and C	3.98	mg/tab.
**HPLC ASSAY**<br>ofi Gingkoflavonglucosides	28.8	mg/tab.
**As**<br>**Pb**<br>**Cd**<br>**Hg**	0.104<br>0.134<br>0.011<br>0.001	ppm<br>ppm<br>ppm<br>ppm
**MICROBIOLOGICAL CONTROL**<br>Eur. Pharm. (2.6.12, 2.6.13 and 2.6.31)<br><br>- **Total Aerobic Microbial Count**<br>- **Total Combined Yeast/Moulds Count**<br>- **Bile-Tolerant Gram-Negative Bacteria**<br>- **Escherichia coli**<br>- **Salmonella**<br>- **Staphylococcus aureus**	 <br><br>500<br>< 10<br>absent<br>absent<br>absent<br>absent	 <br><br>cfu/g<br>cfu/g<br>cfu/g<br>/g<br>/25g<br>/g

Milan, March 31, 2015

Franco Santini

INDENA S.p.A.
MILAN PLANT
TECHNICAL DIRECTOR
Dr. Franco Santini

Dr.ssa Viviana Cerutti
Head of Quality Control
INDENA S.p.A., Milan Plant

Cerutti

indena®

20139 Milano - Viale Ortles, 12 - Tel. 02.57496.1

Manufacturer's Address:
Formulation Development Laboratory
INDENA S.p.A. (Milan Plant)
Viale Ortles, 12
20139 Milan, Italy

STATEMENT – N° 15/036-MI

Object: **Allergens statement**

Product:	**Ginkgo biloba ethanolic extract coated tablets (placebo)**
Indena Code:	**LM42506**
Batch Number:	**89077**
This confirmation form has been completed by:	Name: Dr F. Santini<br>Company: INDENA S.p.A. (Milan plant)<br>Position in the company: Technical Director

We inform that milk and soy derivatives and ingredients containing gluten are included in products manufactured on the same equipment involved in the production of "Ginkgo biloba ethanolic extract coated tablets (placebo)".

Accurate cleaning operations, described in dedicated SOPs, are carried out on the involved equipment before the further manufacturing operations, but at present, on the finished product, no further analytical investigations have been carried out to check the absence of these aforesaid allergenic substances or of other food allergens listed in Annex II of the Regulation 1169/2011/EC.

Milan, April 21, 2015

Franco Santini
INDENA S.p.A.
Dr F. Santini
Technical Director
