# Supplementary material for: Clinical and genomic safety of treatment with Ginkgo biloba L. leaf extract (IDN 5933/Ginkgoselect®Plus) in elderly: a randomised placebo-controlled clinical trial [GiBiEx]
Source: BMC Complement Altern Med. 2018 Jan 22;18:22. doi: 10.1186/s12906-018-2080-5 (PMC5778811; doi:10.1186/s12906-018-2080-5)
Supplement: Supplementary file 2 — Questionnaire. GiBiEx QUESTIONNAIRE. The English version of the questionnaire administered to all participants to the GiBiEx study is reported. (PDF 10 kb) [file 12906_2018_2080_MOESM2_ESM.pdf]

Date Questionnaire ___/___/___ **Participant's ID Number**……………………….

STUDY GiBiEx QUESTIONNAIRE

Please answer the following questions concerning your personal infomations and present health status

PARTICIPANT'S INFORMATION		
Nursing Home ………………………………………………………………………………..		
Date Admission……………………………..	Marital Status ……………………………………	
Ethnicity…………………………………	Education (years)…………………..	
Weight (current)…………Kg<br>Height ………………… m		
Sex M F	Date of Birth___/___/___	Age…………….....

SMOKING HABITS	
**Have you ever smoked ?**	NO Yes EX-Smoker
**If Ex Smoker, How long have you not smoked?**	(from 1-6 months) (from 7-12 months) (more 1 year)

Date Questionnaire ___/___/___ **Participant's ID Number**..........................

**MEDICAL HISTORY**<br><br>***(You can make more than one choice and indicate your desease)***
CARDIOVASCULAR DISEASES:
PATHOLOGIES OF THE BLOOD AND LYMPHATIC SYSTEM:
RESPIRATORY DISEASES:
GASTROINTESTINAL DISEASES:
HEPATIC DISEASES:
DISEASES OF THE GENITOURINARY SYSTEM:
ENDOCRINE, NUTRITIONAL AND METABOLIC DISEASES:
DISEASES OF THE MUSCULOSKELETAL SYSTEM
PSYCHIATRIC DISORDERS:
NEUROLOGICAL DISEASES:
Other:

Date Questionnaire ___/___/___ **Participant's ID Number**..........................

DRUG THERAPY			
**DRUG N.**	**DRUG NAME**	**FREQUENCY OF DRUG ADMINISTRATION**	**PRESCRIPTION DRUG DOSAGE**
**1**			
**2**			
**3**			
**4**			
**5**			
**6**			
**7**			
**8**			
**9**			
**10**			
