# Supplementary material for: Clinical and genomic safety of treatment with Ginkgo biloba L. leaf extract (IDN 5933/Ginkgoselect®Plus) in elderly: a randomised placebo-controlled clinical trial [GiBiEx]
Source: BMC Complement Altern Med. 2018 Jan 22;18:22. doi: 10.1186/s12906-018-2080-5 (PMC5778811; doi:10.1186/s12906-018-2080-5)
Supplement: Supplementary file 3 — Comet Assay Raw data. Individual data relative to comet assay. For each donor data are shown as before (T0) and after (T1) placebo or IDN 5933 administration. (PDF 425 kb) [file 12906_2018_2080_MOESM3_ESM.pdf]

Additional file 3 Comet Assay Raw Data.

Individual data relative to comet assay. For each donor data are shown before (T0) and after (T1) placebo or IDN 5933 administration.

Code number	*Treatment group: Placebo (0) IDN 5933 (1)*	*Blood sampling*	Tail-Length	% TI	Tail Moment	% hedgehogs	*Blood sampling*	Tail-Length	% TI	Tail Moment	% hedgehogs
**5RT**	*0*	*T0*	5.72	3.99	2.74	5.13	*T1*	19.02	16.79	10.58	4.91
**30ST**	*0*	*T0*	12.93	9.05	6.36	8.92	*T1*	3.20	2.58	1.72	0.00
**32ST**	*0*	*T0*	9.32	7.86	5.32	3.77	*T1*	2.85	1.80	1.07	0.59
**33ST**	*0*	*T0*	7.48	5.72	3.78	3.90	*T1*	2.54	1.40	0.80	0.00
**35ST**	*0*	*T0*	12.48	10.54	6.29	0.63	*T1*	7.64	7.19	4.28	1.25
**38ST**	*0*	*T0*	8.14	6.60	4.14	2.45	*T1*	4.23	2.92	2.01	0.00
**39ST**	*0*	*T0*	6.09	5.90	3.89	1.92	*T1*	2.59	1.76	0.84	2.61
**44ST**	*0*	*T0*	6.17	4.77	3.23	2.53	*T1*	10.94	7.52	5.78	0.00
**53MT**	*0*	*T0*	6.68	5.48	3.19	1.85	*T1*	16.19	12.07	7.30	6.54
**55MT**	*0*	*T0*	13.55	9.79	7.32	5.77	*T1*	13.36	10.42	6.49	6.71
**59MT**	*0*	*T0*	20.94	14.09	9.22	2.56	*T1*	17.08	13.56	9.14	3.73
**63MT**	*0*	*T0*	9.53	5.67	3.35	1.85	*T1*	9.37	6.49	4.01	0.64
**65MT**	*0*	*T0*	10.42	5.66	3.43	4.46	*T1*	14.02	10.75	6.63	8.39
**67MT**	*0*	*T0*	14.49	14.43	8.94	4.58	*T1*	13.22	11.60	6.67	0.63
**70MT**	*0*	*T0*	6.80	4.93	3.11	0.00	*T1*	3.75	2.06	1.21	3.16
**72MT**	*0*	*T0*	11.54	8.39	6.00	5.84	*T1*	6.31	3.89	2.41	3.95
**74MT**	*0*	*T0*	19.58	17.69	11.11	4.97	*T1*	8.45	6.27	3.61	2.55
**75MT**	*0*	*T0*	19.63	11.89	8.20	5.16	*T1*	16.34	9.08	5.43	6.41
**77MT**	*0*	*T0*	13.08	9.55	6.39	2.42	*T1*	11.79	8.01	5.35	5.88
**78MT**	*0*	*T0*	7.38	4.10	2.09	2.50	*T1*	9.72	7.04	4.42	3.90
**1RT**	*1*	*T0*	6.02	5.03	2.76	3.21	*T1*	9.27	6.99	4.05	4.35
**10RT**	*1*	*T0*	12.05	10.10	6.73	9.15	*T1*	17.48	15.73	9.81	4.38
**11RT**	*1*	*T0*	5.71	4.34	2.68	3.75	*T1*	6.41	4.83	2.59	3.77
**13RT**	*1*	*T0*	15.02	11.54	6.90	4.52	*T1*	15.25	12.06	8.06	14.19
**14RT**	*1*	*T0*	4.44	3.62	2.09	4.40	*T1*	15.97	13.01	7.88	8.39
**25ST**	*1*	*T0*	4.98	3.98	2.05	3.59	*T1*	1.22	0.63	0.26	1.20
**28ST**	*1*	*T0*	3.32	2.16	1.32	3.07	*T1*	1.64	0.94	0.45	0.00
**29ST**	*1*	*T0*	16.62	14.74	9.40	7.50	*T1*	4.56	2.81	2.33	2.45
**36ST**	*1*	*T0*	5.51	3.44	2.33	4.38	*T1*	2.41	1.59	0.91	1.23
**37ST**	*1*	*T0*	2.44	1.19	0.69	1.31	*T1*	2.81	1.27	0.69	1.30
**40ST**	*1*	*T0*	8.63	8.72	5.52	1.27	*T1*	8.07	6.41	3.76	0.65
**42ST**	*1*	*T0*	8.58	4.23	2.26	1.23	*T1*	2.73	1.64	0.69	0.00
**43ST**	*1*	*T0*	17.67	14.55	9.29	3.73	*T1*	3.87	2.19	0.95	0.00
**51MT**	*1*	*T0*	6.09	4.42	2.68	0.65	*T1*	2.75	1.75	0.77	1.94

**52MT**	*1*	*T0*	10.64	6.07	3.99	3.90	*T1*	10.92	10.76	5.44	2.58
**54MT**	*1*	*T0*	16.83	11.86	8.07	3.21	*T1*	12.97	9.53	6.53	1.92
**56MT**	*1*	*T0*	5.13	3.23	1.72	0.64	*T1*	7.26	5.42	3.50	2.55
**60MT**	*1*	*T0*	18.07	15.94	10.55	5.92	*T1*	9.66	6.43	3.91	2.60
**61MT**	*1*	*T0*	7.34	3.65	2.20	3.03	*T1*	8.23	3.34	1.64	0.66
**62MT**	*1*	*T0*	10.23	6.69	4.19	2.56	*T1*	11.62	7.59	4.60	3.95
**66MT**	*1*	*T0*	5.84	3.86	2.77	1.22	*T1*	10.81	8.19	5.33	1.94
**68MT**	*1*	*T0*	21.19	19.01	12.39	1.99	*T1*	9.01	6.66	4.05	1.90
**71MT**	*1*	*T0*	4.22	2.18	1.51	3.85	*T1*	8.58	4.56	2.77	2.44
**73MT**	*1*	*T0*	10.53	7.63	4.74	4.35	*T1*	11.19	10.03	5.85	7.45
**76MT**	*1*	*T0*	8.90	4.74	2.62	3.75	*T1*	12.86	7.82	4.25	4.91
**79MT**	*1*	*T0*	10.13	6.79	4.47	2.60	*T1*	4.73	2.67	1.85	1.30
**80MT**	*1*	*T0*	13.35	8.54	5.77	2.60	*T1*	10.58	8.08	4.74	4.38
