# Supplementary material for: Clinical and genomic safety of treatment with Ginkgo biloba L. leaf extract (IDN 5933/Ginkgoselect®Plus) in elderly: a randomised placebo-controlled clinical trial [GiBiEx]
Source: BMC Complement Altern Med. 2018 Jan 22;18:22. doi: 10.1186/s12906-018-2080-5 (PMC5778811; doi:10.1186/s12906-018-2080-5)
Supplement: Supplementary file 4 — Gene expression Raw data. Data were analyzed in each sample in terms of increase/decrease of the specific gene expression at T1 compared to T0, with a cut-off value ≥2-fold change (The values reported represent the excel conversion of the original raw data (average Ct calculated out of four replicates loaded onto the 48-wells PCR plate); the raw data are produced as “.csv” file by the EcoIllumina software and need to be converted into excel file for delta delta Ct analysis). (PDF 163 kb) [file 12906_2018_2080_MOESM4_ESM.pdf]

Additional file 4 Summary of the gene expression data carried out in the reported set of patients by real-time PCR analysis (as detailed in the Materials and Methods).

Treatment ID: T, GBLE-treated; PL, placebo-treated

Patient ID: confidential patient identification

Raw Data: Ct average values (qRT-PCR exported data)

Fold change: relative gene expression evaluated at T1 compared to T0

Gene variation: DOWN, gene down-regulation at T1 compared to T0; UP, gene up-regulation at T1 compared to T0; =, non change in gene expression at T1 compared to T0

Treatment ID	Patient ID	Raw Data		Fold change (ΔΔCt method)	Gene variation
		Ct GAPDH	Ct MYB		
T	**11 RT0**	29.24	34.73	1	**DOWN**
	**11 RT1**	28.11	35.3	0.31	
T	**29 ST0**	30.81	36.55	1	**DOWN**
	**29 ST1**	26.07	35.21	0.10	
T	**40 ST0**	26.93	not amplified		
	**40 ST1**	30.15	not amplified		
T	**42 ST0**	28.23	30.12	1	=
	**42 ST1**	28.27	29.73	1.35	
T	**60 MT0**	28.78	31.33	1	**UP**
	**60 MT1**	28.75	29.55	3.36	
T	**66 MT0**	30.58	34.66	1	**UP**
	**66 MT1**	28.27	31.23	2.17	
T	**76 MT0**	29.04	33.68	1	**UP**
	**76 MT1**	26.83	29.06	5.31	
T	**62 MT0**	31.97	33.15	1	**DOWN**
	**62 MT1**	29.4	32.55	0.26	
PL	**65 MT0**	30.84	34.06	1	**UP**
	**65 MT1**	31.7	32.94	3.94	
PL	**30 ST0**	32.38	37.19	1	**DOWN**
	**30 ST1**	27.7	34.49	0.25	
PL	**35 ST0**	34.22	39.34	1	**DOWN**
	**35 ST1**	27.89	35.7	0.15	
PL	**44 ST0**	29.54	31.27	1	**DOWN**
	**44 ST1**	25.82	29.7	0.23	
PL	**53 MT0**	29.86	30.5	1	=
	**53 MT1**	26.53	27.52	0.78	
PL	**55 MT0**	21.75	22.8	1	=
	**55 MT1**	29.1	29.21	1.92	
PL	**72 MT0**	27.56	27.66	1	=
	**72 MT1**	32.46	32.37	1.14	
PL	**74 MT0**	26.64	27.91	1	=
	**74 MT1**	29.06	30.76	0.74	
PL	**59 MT0**	28.88	30.95	1	**DOWN**
	**59 MT1**	29.01	32.51	0.37	

Treatment ID	Patient ID	Raw Data		Fold change (ΔΔCt method)	Gene variation
		Ct GAPDH	Ct P53		
T	**11 RT0**	29.24	36.49	1	=
	**11 RT1**	28.11	34.53	1.78	
T	**29 ST0**	30.81	38.4	1	**UP**
	**29 ST1**	26.07	32.19	2.77	
T	**40 ST0**	26.93	36.27	1	**UP**
	**40 ST1**	30.15	34.88	24.42	
T	**42 ST0**	28.23	40	1	**UP**
	**42 ST1**	28.27	34	65.80	
T	**60 MT0**	28.78	not amplified		
	**60 MT1**	28.75	not amplified		
T	**66 MT0**	30.58	39.63	1	**UP**
	**66 MT1**	28.27	36.02	2.46	
T	**76 MT0**	29.04	36.13	1	**DOWN**
	**76 MT1**	26.83	36.09	0.22	
T	**62 MT0**	31.97	25.32	1	**DOWN**
	**62 MT1**	29.4	25.52	0.15	
PL	**65 MT0**	30.84	29.1	1	**UP**
	**65 MT1**	31.7	24.85	34.54	
PL	**30 ST0**	32.38	40	1	**UP**
	**30 ST1**	27.7	32.67	6.28	
PL	**35 ST0**	34.22	38.54	1	=
	**35 ST1**	27.89	33.07	0.55	
PL	**44 ST0**	29.54	40	1	=
	**44 ST1**	25.82	35.68	1.52	
PL	**53 MT0**	29.86	35.08	1	**DOWN**
	**53 MT1**	26.53	35.08	0.10	
PL	**55 MT0**	21.75	not amplified		
	**55 MT1**	29.1	not amplified		
PL	**72 MT0**	27.56	not amplified		
	**72 MT1**	32.46	not amplified		
PL	**74 MT0**	26.64	not amplified		
	**74 MT1**	29.06	not amplified		
PL	**59 MT0**	28.88	26.5	1	=
	**59 MT1**	29.01	27.23	0.66	

Treatment ID	Patient ID	Raw Data		Fold change (ΔΔCt method)	Gene variation
		Ct GAPDH	Ct CTNNB1		
T	**11 RT0**	29.24	not amplified		
	**11 RT1**	28.11	not amplified		
T	**29 ST0**	30.81	40	1	**DOWN**
	**29 ST1**	26.07	39.81	0.043	
T	**40 ST0**	26.93	not amplified		
	**40 ST1**	30.15	not amplified		
T	**42 ST0**	28.23	not amplified		
	**42 ST1**	28.27	not amplified		
T	**60 MT0**	28.78	not amplified		
	**60 MT1**	28.75	not amplified		
T	**66 MT0**	30.58	not amplified		
	**66 MT1**	28.27	not amplified		
T	**76 MT0**	29.04	not amplified		
	**76 MT1**	26.83	not amplified		
T	**62 MT0**	31.97	32.11	1	**DOWN**
	**62 MT1**	29.4	38.09	0.003	
PL	**65 MT0**	30.84	not amplified		
	**65 MT1**	31.7	not amplified		
PL	**30 ST0**	32.38	40	1	**UP**
	**30 ST1**	27.7	37.83	0.18	
PL	**35 ST0**	34.22	40	1	=
	**35 ST1**	27.89	36.84	0.11	
PL	**44 ST0**	29.54	not amplified		
	**44 ST1**	25.82	not amplified		
PL	**53 MT0**	29.86	not amplified		
	**53 MT1**	26.53	not amplified		
PL	**55 MT0**	21.75	31.5	1	**DOWN**
	**55 MT1**	29.1	40	0.45	
PL	**72 MT0**	27.56	not amplified		
	**72 MT1**	32.46	not amplified		
PL	**74 MT0**	26.64	not amplified		
	**74 MT1**	29.06	not amplified		
PL	**59 MT0**	28.88	not amplified		
	**59 MT1**	29.01	not amplified		
