# Supplementary material for: Clinical and genomic safety of treatment with Ginkgo biloba L. leaf extract (IDN 5933/Ginkgoselect®Plus) in elderly: a randomised placebo-controlled clinical trial [GiBiEx]
Source: BMC Complement Altern Med. 2018 Jan 22;18:22. doi: 10.1186/s12906-018-2080-5 (PMC5778811; doi:10.1186/s12906-018-2080-5)
Supplement: Supplementary file 5 — Micronucleus Assay Raw data. Individual data relative to the MN Assay. Data are reported as before (T0) and after (T1) placebo or IDN 5933 administration. (PDF 683 kb) [file 12906_2018_2080_MOESM5_ESM.pdf]

Additional file 5 Micronucleus Assay raw data

Individual data relative to MN Assay, before (T0) and after (T1) placebo or IDN 5933 administration.

Data MN T0

		T0																		
		Replicate A1		Replicate B1		Replicate A2		Replicate B2		Replicate A3		Replicate B3		Replicate A4		Replicate B4		Total BNC	Total MN	MN ‰
Patient Code	Status_Treated_T_Placebo_P	BNC	MN	BNC	MN	BNC	MN	BNC	MN	BNC	MN	BNC	MN	BNC	MN	BNC	MN			
1R	T	15	0	0	0	18	0	0	0	13	1	2	0					48	1	
5R	P	21	1	21	0	14	0	195	1	6	0	32	0					289	2	6.92
10R	T	214	3	105	0	187	2	64	1	45	0	4	0					619	6	9.69
11R	T	100	2	61	1	78	1	39	1	3	0	31	0					312	5	16.03
13R	T	888	5	513	6													1401	11	7.85
14R	T	639	4	836	6													1475	10	6.78
51M	T	415	6	770	6													1185	12	10.13
52M	T	130	1	342	2	217	1	457	3									1146	7	6.11
53M	P	371	3	390	0	326	2	317	4									1404	9	6.41
54M	T	138	3	94	2	274	4	147	2	15	2	66	2					734	15	20.44
55M	P	203	0	286	1	214	2	267	4	161	1	97	0					1228	8	6.51
56M	T	4	0	213	1	150	1	309	3	32	0	117	1					825	6	7.27
59M	P	254	1	117	2	362	4	177	0	89	0	26	1					1025	8	7.80

60M	T	249	1	282	1	231	2	301	2									1063	6	5.64
61M	T	442	5	293	4	316	6	310	1									1361	16	11.76
62M	T	606	3	618	0													1224	3	2.45
63M	P	814	3	455	1													1269	4	3.15
65M	P	262	5	229	2	41	0	247	7	163	0	29	0					971	14	14.42
66M	T	175	3	307	0	188	1	189	1	9	0	194	0					1062	5	4.71
67M	P	513	3	428	4	471	0	399	0									1811	7	3.87
68M	T	0	0	184	1	203	4	267	5	10	0							664	10	15.06
70M	P	421	2	357	5	419	0	443	1									1640	8	4.88
71M	T	127	0	518	2	265	4	511	6									1421	12	8.44
72M	P	135	4	576	14	181	2	462	4									1354	24	17.73
73M	T	426	3	232	1	127	0	189	0	131	0	14	0					1119	4	3.57
74M	P	210	4	265	5	216	5	684	6									1375	20	14.55
75M	P	543	2	392	2	300	3	583	4									1818	11	6.05
76M	T	361	2	373	5	472	1	617	4									1823	12	6.58
77M	P	161	2	157	1	79	1	118	2	120	0	44	0					679	6	8.84
78M	P	223	3	481	4	365	3	415	7									1484	17	11.46
79M	T	79	1	516	6	351	8	354	10									1300	25	19.23
80M	T	244	0	489	7	722	2	433	0									1888	9	4.77
25S	T	557	11	209	3	627	11	349	1									1742	26	14.93
28S	T	495	2	425	2	747	3	543	5									2210	12	5.43
29S	T	458	5	714	11													1172	16	13.65
30S	P	531	3	735	4													1266	7	5.53

32S	P	174	4	311	3	34	0	274	2	132	3	82	1					1007	13	12.91
33S	P	465	4	238	3	391	1	281	0									1375	8	5.82
35S	P	14	0	8	0	285	0	299	7	11	1	139	2					756	10	13.23
36S	T	219	14	260	5	306	5	306	5									1091	29	26.58
37S	T	259	1	716	7	293	0	584	4									1852	12	6.48
38S	P	7	0	2	0	290	2	419	4	90	0	153	4					961	10	10.41
39S	P	160	2	175	2	146	1	158	1	127	0	166	0					932	6	6.44
40S	T	342	4	202	1	168	2	127	3	98	0	142	3					1079	13	12.05
42S	T	290	3	477	5	266	1	399	4									1432	13	9.08
43S	T	243	4	124	2	85	2	70	0	60	1	92	0					674	9	13.35
44S	P	389	5	581	14	401	6	530	15									1901	40	21.04

Data MN_T1

		T1																		
		Replicate A1		Replicate B1		Replicate A2		Replicate B2		Replicate A3		Replicate B3		Replicate A4		Replicate B4		Total BNC	Total MN	MN‰
**Patient Code**	**Status_Treated_T_Placebo_P**	**BNC**	**MN**	**BNC**	**MN**	**BNC**	**MN**	**BNC**	**MN**	**BNC**	**MN**	**BNC**	**MN**	**BNC**	**MN**	**BNC**	**MN**			
1R	T	718	3	1393	40													2111	43	20,36949
5R	P	486	8	580	11													1066	19	17,82364
10R	T	424	6	589	9													1013	15	14,8075
11R	T	774	16	862	11													1636	27	16,50367
13R	T	614	13	844	13													1458	26	17,83265
14R	T	492	1	453	1	357	1	388	1									1690	4	2,366864
51M	T	348	3	285	1	269	4	149	0									1051	8	7,611798
52M	T	294	2	252	2	40	0	154	0	1	1	102	1					843	6	7,117438
53M	P	632	5	545	4													1177	9	7,646559
54M	T	260	5	311	6	221	7	274	5									1066	23	21,57598
55M	P	547	4	0	0	649	10	0	0									1196	14	11,70569
56M	T	229	1	290	1	195	0	195	0	323	1	385	1					1617	4	2,473717
59M	P	0	0	931	4	926	14	911	8									2768	26	9,3930

																				64
60M	T	531	11	515	13													1046	24	22,94455
61M	T	326	4	318	5	281	2	475	2									1400	13	9,285714
62M	T	347	2	419	2	364	2	355	0									1485	6	4,040404
63M	P	530	8	512	10													1042	18	17,27447
65M	P	237	4	186	9	194	3	232	6	3	0	91	0					943	22	23,3298
66M	T	132	1	74	0	129	0	129	1	113	2	331	0					908	4	4,405286
67M	P	389	3	129	1	201	1	219	2	608	3	334	3					1880	13	6,914894
68M	T	357	8	321	5	246	6	263	6									1187	25	21,0615
70M	P	155	1	216	2	160	0	322	3	135	1	42	0					1030	7	6,796117
71M	T	515	9	758	8													1273	17	13,35428
72M	P	990	5	165	3													1155	8	6,926407
73M	T	254	6	247	2	395	7	260	3									1156	18	15,57093
74M	P	673	18	583	15													1256	33	26,27389
75M	P	477	1	449	6	573	2	629	6									2128	15	7,048872
76M	T	591	4	953	12													1544	16	10,36269
77M	P	38	0	187	5	349	5	345	3	10	0	1	0					930	13	13,97849
78M	P	713	13	467	12													1180	25	21,186

																				44
79M	T	171	4	797	11	540	9	613	8									2121	32	15,08722
80M	T	53	0	56	0	13	0	39	0	15	1	73	0					249	1	4,016064
25S	T	278	1	776	9													1054	10	9,487666
28S	T	255	2	650	10	88	0	699	1									1692	13	7,683215
29S	T	299	3	692	6													991	9	9,081736
30S	P	516	0	633	3													1149	3	2,610966
32S	P	462	3	307	5	396	0	338	2									1503	10	6,65336
33S	P	329	1	769	2													1098	3	2,73224
35S	P	598	9	634	10													1232	19	15,42208
36S	T	201	3	433	9	85	0	364	3									1083	15	13,85042
37S	T	283	6	722	13													1005	19	18,90547
38S	P	105	4	5	0	83	0	51	0	85	0	15	1					344	5	14,53488
39S	P	298	5	485	6	319	3	241	2									1343	16	11,91363
40S	T	135	2	372	7	69	0	364	4	12	0	82	0					1034	13	12,57253
42S	T	271	2	136	2	110	1	312	1	36	0	420	12					1285	18	14,00778
43S	T	66	1	22	0	92	1	73	0	1	0	217	1					471	3	6,369427
44S	P	33	1	35	0	26	0	37	0	32	1					168	0	331	2	6,0422

																					96
